# Compact 20-W GaN Internally Matched Power Amplifier for 2.5 GHz to 6 GHz Jammer Systems

**DOI:** 10.3390/mi11040375

**Published:** 2020-04-02

**Authors:** Min-Pyo Lee, Seil Kim, Sung-June Hong, Dong-Wook Kim

**Affiliations:** Department of Radio and Information Communications Engineering, Chungnam National University, Daejeon 34134, Korea; dignitymp20@naver.com (M.-P.L.); ksl4896@naver.com (S.K.); hsj_1006@naver.com (S.-J.H.)

**Keywords:** wideband, GaN, HEMT, power amplifier, jammer system

## Abstract

In this paper, we demonstrate a compact 20-W GaN internally matched power amplifier for 2.5 to 6 GHz jammer systems which uses a high dielectric constant substrate, single-layer capacitors, and shunt/series resistors for low-Q matching and low-frequency stabilization. A GaN high-electron-mobility transistor (HEMT) CGH60030D bare die from Wolfspeed was used as an active device, and input/output matching circuits were implemented on two different substrates using a thin-film process, relative dielectric constants of which were 9.8 and 40, respectively. A series resistor of 2.1 Ω was chosen to minimize the high-frequency loss and obtain a flat gain response. For the output matching circuit, double λ/4 shorted stubs were used to supply the drain current and reduce the output impedance variation of the transistor between the low-frequency and high-frequency regions, which also made wideband matching feasible. Single-layer capacitors effectively helped reduce the size of the matching circuit. The fabricated GaN internally matched power amplifier showed a linear gain of about 10.2 dB, and had an output power of 43.3–43.9 dBm (21.4–24.5 W), a power-added efficiency of 33.4–49.7% and a power gain of 6.2–8.3 dB at the continuous-wave output power condition, from 2.5 to 6 GHz.

## 1. Introduction

GaN high-electron-mobility transistor (HEMT) wideband power amplifiers have been studied for multi-mode communication systems, electronic warfare systems, and other frequency-agile systems that required high-power operation over a wide frequency range [1,2,3,4,5,6,7,8,9,10]. While the transistor gain decreases with the frequency, a wideband power amplifier requires a flat gain performance in the interested bandwidth. In addition, the inherent reactance of the transistor, mainly due to its drain-to-source capacitance, limits the frequency bandwidth of the power amplifier as the Bode–Fano gain-bandwidth product describes [11,12]. To overcome this limitation, a multiple inductor-capacitor (LC) ladder configuration may be a proper choice, but it is area-inefficient and degrades the cost competitiveness, especially for expensive GaN monolithic microwave integrated circuits.

In this paper, we present a compact 20-W internally matched power amplifier for 2.5 to 6 GHz electronic warfare jammer applications which uses a GaN HEMT device and can be integrated into a small metal package. The novelty of this work originates in a practical combination of a series resistor, a shunt resistor-capacitor (RC)sub-circuit, a high dielectric constant matching substrate, and single-layer capacitors to maximize the size reduction effect. Input and output matching circuits for the power amplifier are implemented on two different thin-film substrates with the relative dielectric constants of 9.8 and 40, and, as a part of the matching circuit, single-layer capacitors and thin-film shunt/series resistors are utilized to achieve the circuit stabilization and flat gain, in addition to its compact size.

## 2. Power Amplifier Design

### 2.1. Device Description

**Figure 1:**
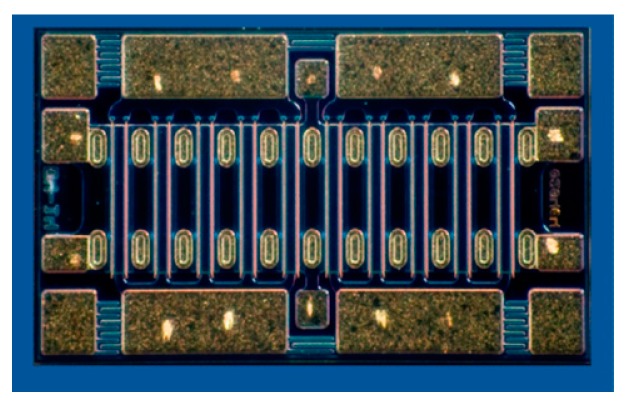
Photograph of a GaN high-electron-mobility transistor (HEMT) bare die (CGH60030D) from Wolfspeed.

**Figure 2:**
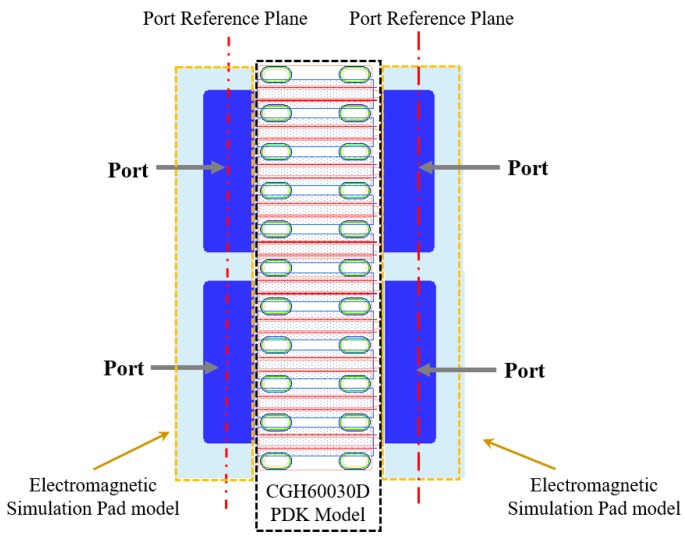
Modified large-signal transistor model with a foundry process design kit model and electromagnetic simulation pad models.

The GaN HEMT bare die with the 0.4 μm gate length (CGH60030D, Wolfspeed, Inc., Research Triangle Park, NC, USA) that is provided by Wolfspeed has a typical power density of 4.5 W/mm and a maximum output power of 30 W at the drain pad and is applicable up to 6 GHz. Considering the loss of the matching circuit itself and the impedance mismatch loss, CGH60030D is a proper choice for the output power of more than 20 W from 2.5 to 6 GHz. Figure 1 and Table 1 show the photograph and device parameters of CGH60030D [13]. The transistor bare die consists of two 10 × 360 μm cells and has four pads on the gate and drain sides. Because the large-signal model provided by the manufacturer has only two ports for the gate and drain pads, it is difficult to fully include the bonding wire effect and the phase balance on each pad. In our work, we use a modified large-signal model combining the transistor unit cell model, which is based on the manufacturer’s foundry process, with three-dimensional electromagnetic simulation models of the gate/drain pads and bonding wires [14,15]. Figure 2 shows the modified large-signal transistor model, the port reference planes of which are on the middle of its gate and drain pads to consider practical wire bonding effects effectively.

### 2.2. Input and Output Matching Circuit Design

We simulated the GaN HEMT under the bias conditions of V_DS_ = 28 V and I_DS_ = 250 mA, and predicted small-signal input impedances at 2.5 GHz and 6 GHz as Z_S,2.5 GHz_ = 1.39 + j2.11 Ω and Z_S,6 GHz_ = 0.89 + j0.11 Ω. Because the transistor was unstable below 7.9 GHz, we performed source-pull and load-pull simulations after stabilizing the transistor with shunt and series resistors. With the drain pad effect eliminated, the transistor was simulated up to the third harmonic by a load-pull tuner [16,17]. The simulation results showed that the optimum load impedances at 2.5 GHz and 6 GHz were Z_L,2.5 GHz_ = 8.00 + j4.91 Ω and Z_L,6 GHz_ = 5.41 + j3.86 Ω, respectively.

**Figure 3:**
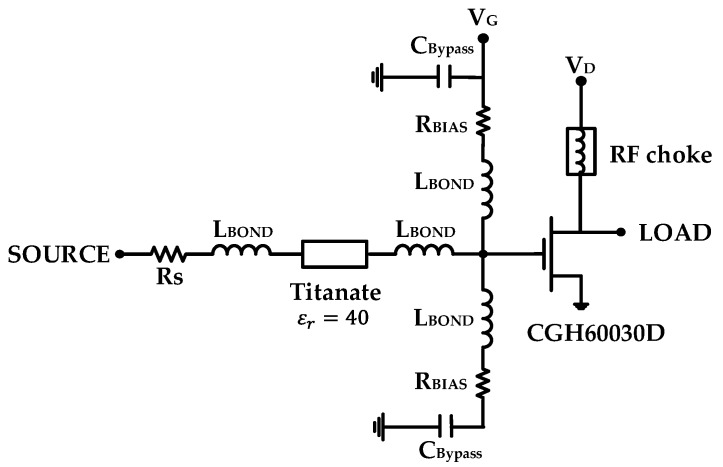
GaN HEMT with the stabilization circuit of the series and shunt resistors.

Figure 3 shows a schematic circuit diagram of the GaN HEMT with the stabilization circuit. To secure the low-frequency stability of the transistor and apply the gate bias voltage through the resistor, the shunt circuit of the resistor R_BIAS_ and the capacitor C_Bypass_ was inserted between the transistor and the input matching circuit. The element values of the shunt circuit were determined to be C_Bypass_ = 39 pF and R_BIAS_ = 36 Ω to simultaneously achieve stability and low loss.

Because the high-frequency gain of the transistor is typically lower than its low-frequency gain, the high-frequency loss of the stabilization circuit should be lower than the circuit’s low-frequency loss. However, because the input impedance of the transistor is very low, low-loss impedance matching is very difficult in a wide frequency range only with the simple matching circuit of a few LC elements. To compromise the low loss and compact size, we used a titanate substrate with the high relative dielectric constant of 40, and applied a series resistor of R_S_ = 2.1 Ω which made the stability factor k more than 1 and a flat gain in the interested bandwidth by increasing the low-frequency loss and reducing the high-frequency loss. 

**Figure 4:**
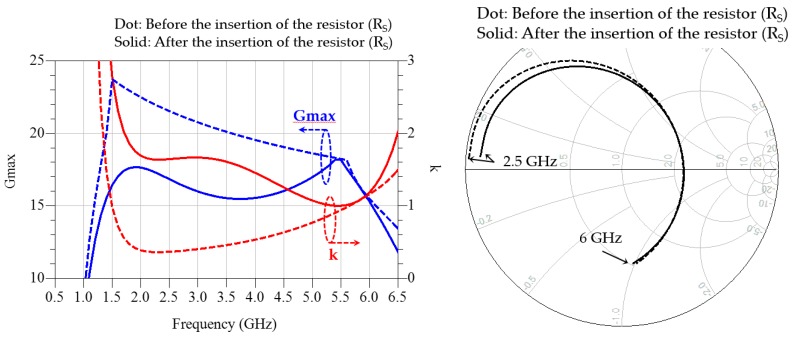
Variation of the stability factor k, maximum available gain (G_max_), and input impedance after the insertion of the series resistor R_S_, with the shunt resistor attached.

Figure 4 shows the variation of the stability factor k, maximum available gain (G_max_), and input impedance before and after the insertion of the series resistor R_S_. As the dotted impedance trace on the Smith chart in Figure 4 implies, the titanate substrate and bonding wires spread the input impedance trace of the transistor greatly in both of the low-frequency region and the high-frequency region, which maintains the low-frequency impedance as very low, and moves the high-frequency impedance to a high-value region. Therefore, a small series resistor greatly reduces the low-frequency gain, and, by contrast, does not affect the high-frequency gain because of the frequency-dependent voltage-dividing ratio.

**Figure 5:**
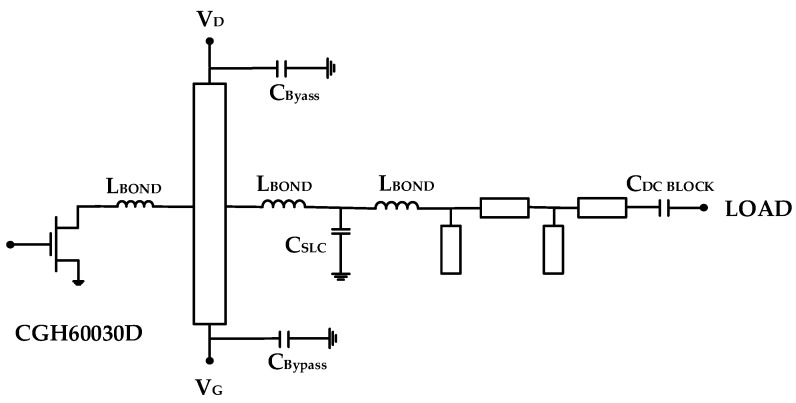
Output matching circuit for the optimum load impedance.

**Figure 6:**
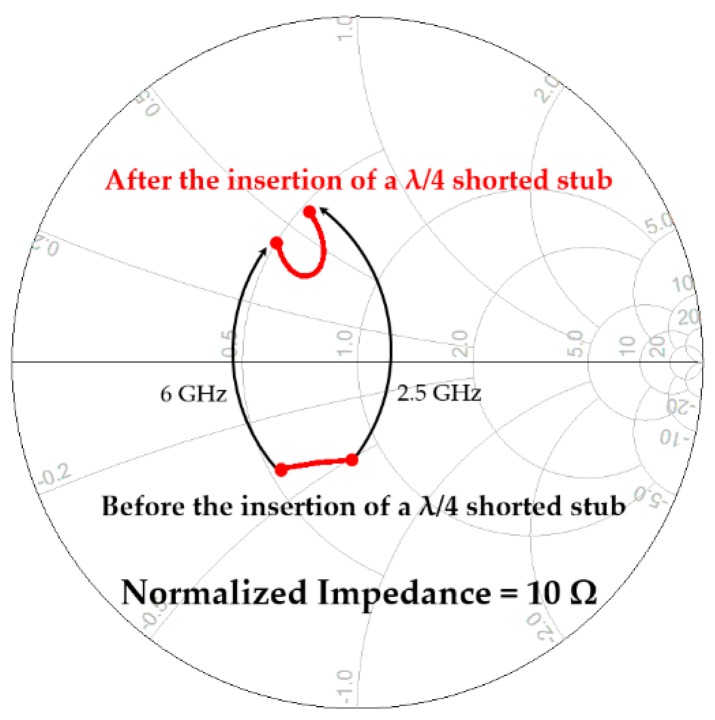
Variation of the transistor’s output impedance before and after the insertion of λ/4 shorted stubs.

Figure 5 shows the output matching circuit that is tuned to the optimum load impedance. Double λ/4 shorted stubs were used for a DC drain current supply and had an LC-parallel resonator effect at the center frequency, thus reducing the variation of the transistor’s output impedance trace and facilitating wideband impedance-matching within a low-Q region on the Smith chart [18,19]. Figure 6 shows the variation of the output impedance trace before and after the insertion of the λ/4 shorted stubs. The output impedance trace was extracted as we saw the drain of the transistor at the bias line position. A single-layer capacitor followed the λ/4 shorted stubs, which resulted in the size reduction of the output matching circuit, although multiple thin-film substrates complicated the assembly process a little. A cascaded low-pass LC network was used for fine tuning of the desired output impedance, and was implemented on an alumina substrate.

**Figure 7:**
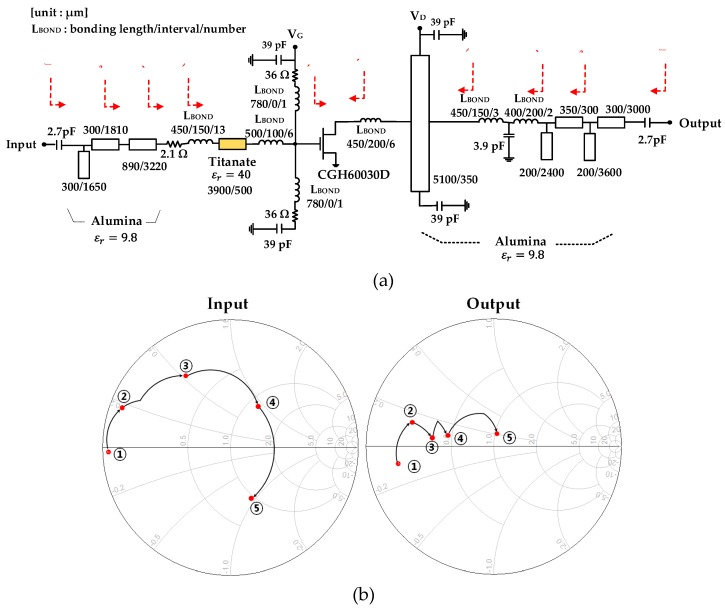

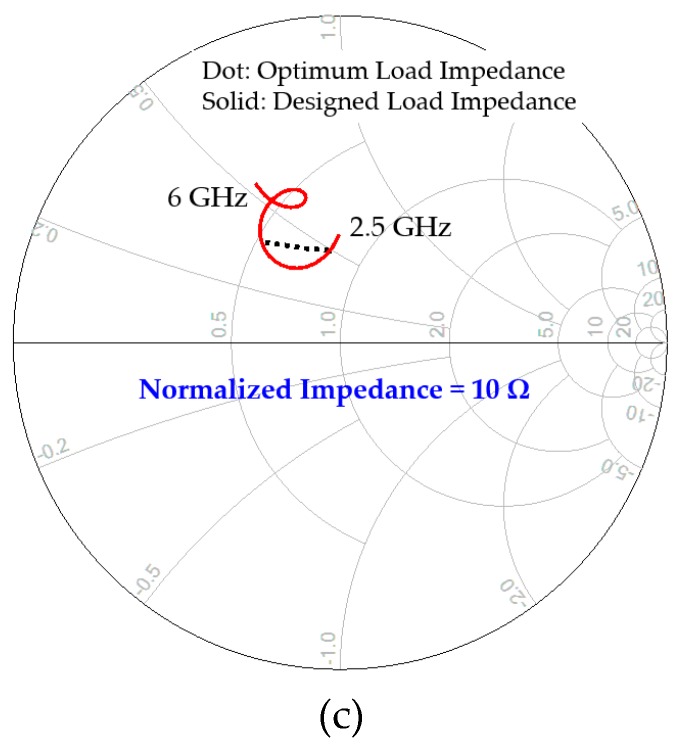
Schematic circuit diagram of the designed power amplifier (**a**), input/output matching impedance traces at 4 GHz (**b**), and the designed load impedance trace seen from the drain of the transistor (**c**).

Figure 7 shows a schematic circuit diagram of the designed power amplifier, exemplary input/output impedance matching traces at 4 GHz, and the designed load impedance trace seen from the drain of the transistor. The input impedance was not well matched because of the very low input impedance of the transistor. On the other hand, the output impedance showed a reasonably good matching. To improve the input matching condition, we should use lossy matching or multiple LC matching techniques, but the former degrades the gain performance, and the latter increases the circuit size. An insufficient gain margin makes it difficult to choose lossy matching, and the large circuit size makes compact integration into a small standard metal package infeasible. Therefore, the input matching properly compromised with the size of the input matching circuit because the input mismatch could be improved with the use of the balanced amplifier configuration [20,21,22].

## 3. Fabrication and Measurement

### 3.1. Power Amplifier Fabrication

**Figure 8:**
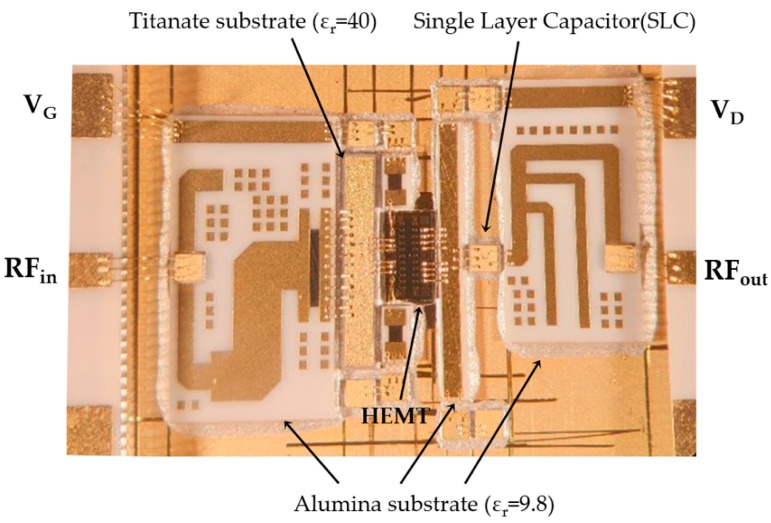
Fabricated internally matched power amplifier using a GaN HEMT bare die, input/output thin-film matching substrates, and single-layer capacitors (circuit area = 9.9 mm × 6.8 mm).

Figure 8 shows a fabricated internally matched power amplifier. Input and output matching circuits were implemented on alumina substrates and a titanate substrate using a thin-film process [23]. The GaN HEMT bare die was attached onto a CPC (Cu/Mo70Cu/Cu) carrier with high thermal conductivity using an AuSn (80/20) eutectic process. The complete circuit occupied 9.9 mm × 6.8 mm on the carrier. The GaN HEMT bare die, single-layer capacitors, input/output matching circuits, and microstrip feed lines on RO4003C (Rogers Corporation, Chandler, AZ, USA) for microwave testing were interconnected by 1 mil Au wedge bonding wires.

### 3.2. Power Amplifier Measurement

**Figure 9:**
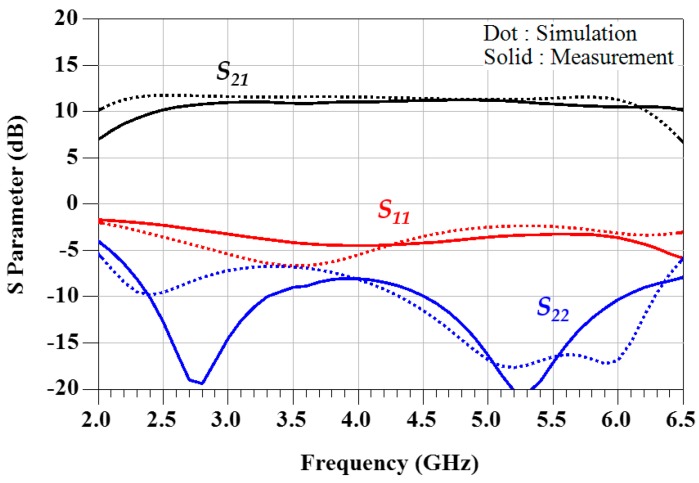
Simulated and measured S-parameter results (simulation: dotted lines, measurement: solid lines).

The fabricated power amplifier was measured on a heat-sinking jig under the bias conditions of V_DS_ = 28 V and I_DS_ = 250 mA. The measured S-parameter data were compared with the simulated data in Figure 9. The measured results showed a linear gain of more than 10.2 dB and a return loss of more than 2.3 dB from 2.5 to 6 GHz. The low-frequency gain decreased slightly, and the roll-off of the high-frequency gain moved to about 6.5 GHz due to the minute change of the implemented load impedance after the device assembly. 

**Figure 10:**
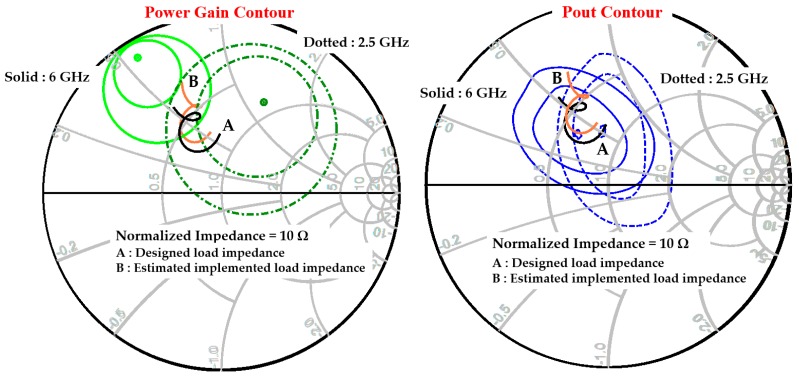
Designed and implemented load impedance traces with the power gain contours and the output power contours at 2.5 GHz and 6 GHz.

Figure 10 shows the designed load impedance trace and implemented load impedance trace estimated after the device assembly on Smith charts, together with the power gain contours and the output power contours at 2.5 GHz and 6 GHz. As shown in Figure 10, the small shift of the implemented load impedance from the designed load impedance slightly increased the high-frequency gain.

**Figure 11:**
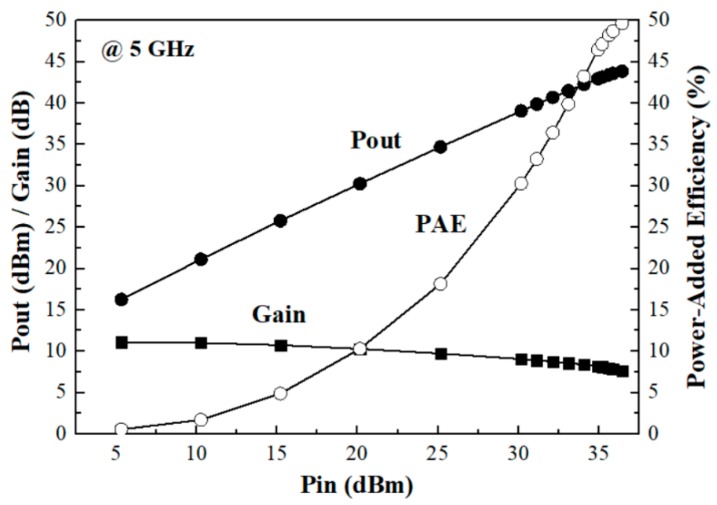
Output power, power gain, and power-added efficiency (PAE) across the input power at 5 GHz.

Figure 11 shows the output power, power gain, and power-added efficiency (PAE) of the fabricated power amplifier across the input power at 5 GHz where the maximum PAE was measured. The power gain was decreased from 11.1 to 7.6 dB with the varying input power, which corresponded to 3.5 dB power compression. The saturated output power was 43.9 dBm, and the power-added efficiency was 49.7% at the power saturation condition.

**Figure 12:**
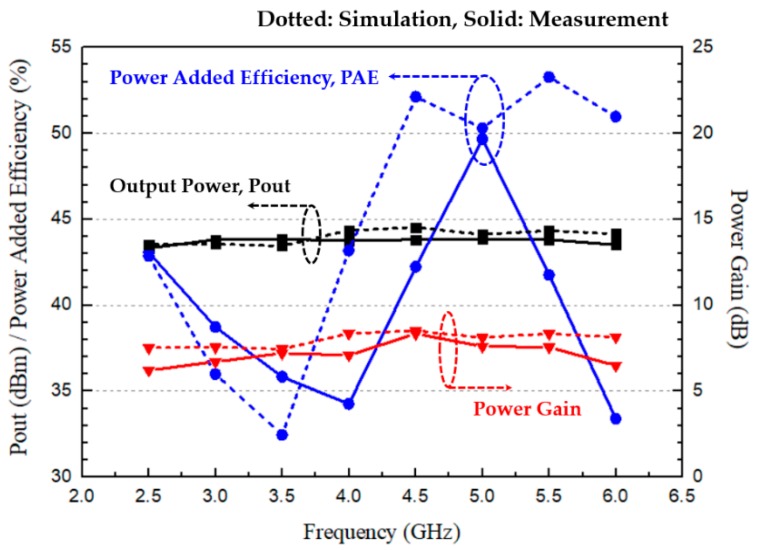
Measured continuous-wave output power performance of the fabricated power amplifier at V_DS_ = 28 V and I_DS_ = 250 mA in the frequency range of 2.5–6 GHz under the power saturation condition.

Figure 12 shows the measured continuous-wave output power performance of the power amplifier under the bias conditions of V_ds_ = 28 V and I_ds_ = 250 mA. The measurement was done from 2.5 to 6 GHz with a 0.5 GHz step frequency. The measured results showed that the amplifier had an output power of 43.3–43.9 dBm, a power-added efficiency (PAE) of 33.4–49.7%, and a power gain of 6.2–8.3 dB. The maximum deviation of the output power was 0.7 dB at 4.5 GHz, and the power-added efficiency (PAE) was slightly degraded due to some mismatch of the designed load impedance and the implemented load impedance after the device assembly. Our simulations estimated an insertion loss of 0.3–0.52 dB for the output matching circuit itself across 2–6 GHz [24]. The power gain decreased at both ends of the designed bandwidth with the maximum 1.5 dB, which was caused by slightly earlier compression with the increase in the input power, as predicted from the trace comparison of the designed load impedance and the implemented load impedance on the output power contours in Figure 10.

Table 2 compares our measured power performance with other published results, showing that our work is very competitive in terms of the bandwidth, output power, and power-added efficiency, despite the compact size.

## 4. Conclusions

We presented the compact 20-W GaN internally matched power amplifier operating from 2.5 to 6 GHz that utilizes a GaN HEMT bare die, single-layer capacitors, and thin-film substrates for input and output matching circuits. The fabricated power amplifier achieved stable operation and flat gain performance by using shunt and series resistors. Under the continuous-wave output power condition, it showed the output power of 43.3–43.9 dBm (21.4–24.5 W), the PAE of 33.4–49.7% and the power gain of 6.2–8.3 dB in the frequency range of 2.5–6 GHz. The developed power amplifier will be effectively used in wireless and military applications that require high output power over a wide frequency range.

## Figures and Tables

**Table 1:** Device parameters of CGH60030D.

Parameters	Specifications
operating frequency	DC—6 GHz
saturated output power	30 W
power-added efficiency	65% at 4 GHz
small-signal gain	15 dB at 4 GHz
operating voltage	28 V
size	920 μm × 1660 μm

**Table 2:** Comparison of our work and previously published wideband GaN HEMT power amplifiers.

References	Frequency [GHz]	Power Gain [dB]	P_out_ [dBm]	PAE [%]	Technology	Drain Voltage [V]	Size [mm^2^]
Ref. [25]	1.1–2.7	9.5–11.5	43–45	59–72	PCB	28	120 × 50
Ref. [26]	2–6	8–9	43.5–44.5	40–47	PCB	30	est. 68 × 28
Ref. [27]	1.7–3	9.8–10.7	43.8–44.4	57.2–71.1 **^1^**	PCB	28	est. 75 × 35
Ref. [28]	0.3–2.3	≥10	40–43.5	58–69	PCB	28	59 × 50
Ref. [29]	0.6–3.8	9–14	40–42	46–75	PCB	28	69 × 40
Ref. [30]	2–4	≥9.8	44	37–52^1^	Quasi-MMIC	50	≤420
This work	2.5–6	6.2–8.3	43.3–43.9	33.4–49.7	Thin film	28	9.9 × 6.8

**^1^**: drain efficiency; est.: estimated.
